# Synthesis of a Stable and High-Concentration BaHf_x_Ti_1−x_O_3_ Sol–Gel for High Electromechanical Performance of Bulk Ceramics

**DOI:** 10.3390/ma16237452

**Published:** 2023-11-30

**Authors:** Damien Brault, Thomas Richardot, Philippe Boy, Philippe Belleville, Franck Levassort, Maxime Bavencoffe

**Affiliations:** 1 GREMAN UMR 7347, INSA–CVL, University of Tours, CNRS, 37200 Tours, France; damien.brault@univ-tours.fr (D.B.); t.richardot@laposte.net (T.R.); franck.levassort@univ-tours.fr (F.L.); 2 CEA, DAM, Le Ripault, 37260 Monts, France; philippe.boy@cea.fr (P.B.); philippe.belleville@cea.fr (P.B.)

**Keywords:** piezoelectricity, lead-free, sol–gel synthesis, barium titanate, ceramics

## Abstract

Lead-based materials are widely used in piezoceramics due to their high electromechanical properties. However, due to environmental protection and sustainable development, the use of the toxic element lead (Pb) in electronic devices is strictly restricted, therefore requiring the rapid development of piezoelectric-based devices with lead-free ceramics. In this context, a lead-free doped barium titanate was studied with a dual objective. First, a new sol–gel method to synthesize Hf^4+^-doped BaHf_x_Ti_1−x_O (BHT) with x = 0.05, 0.075, and 0.10 is presented. Such BHT sols were prepared at high concentrations of up to 1 M. Dilution in ethylene glycol allowed parameters (viscosity, colloid sizes, etc.) to be controlled, which ensured a time-stable sol for several months at room temperature. Second, densified bulk ceramics with attrited powders were obtained from these sols and showed very good electromechanical properties, with a thickness coupling factor of *k_t_* = 47% (BaHf_0.05_Ti_0.95_O_3_ sintered at 1500 °C/6 h). These results are a first step that will allow the processing of lead-free piezoelectric thick films using a sol–gel composite method for vibrational energy harvesting applications.

## 1. Introduction

Perovskite-based ferroelectric materials have attracted increasing interest in recent decades in piezoelectric device applications such as capacitors, memory storage devices, actuators, and transducers [1,2,3,4,5,6,7]. Currently, lead-based perovskites (PZTs) are commonly used for their high electromechanical performance (the thickness coupling coefficient *k_t_* = 0.48 for a standard PZT) [8]. However, the toxicity of lead that these materials contain has increased the need to develop lead-free materials. Today, many countries around the world want to limit or even ban the use of lead in devices [9]. Great efforts have been made, not only in Europe but also in many countries around the world, to develop efficient lead-free piezoelectric materials. In particular, so-called lead-free “vibrational energy harvesters” have gained interest, as they can convert any form of mechanical energy into electricity due to their piezoelectric properties. This technology requires the development of piezoelectric thick films (with thicknesses of a few tens of micrometers) with the original shape of a bimorph cantilever, allowing operation at low frequencies, which makes the expansion of this technology more difficult [10]. In this context, sodium niobate (NN) and barium titanate (BT) systems have been widely investigated [11].

Among these promising functional materials, BaTiO_3_ (BT) was the first ferroelectric material studied [12,13]. This composition greatly contributed to studying the fundamental bases of piezoelectricity [14]. Adjusting the nature and concentration of the perovskite dopant can facilitate its phase transition to a desired structure, hence enhancing its corresponding dielectric and ferroelectric properties. However, the Curie temperature (T_C_, i.e., the ferro/para-electric transition temperature) is lowered with the doping rate [15,16]. In this framework, many BaTiO_3_ dopants have been investigated, such as Sn, Zr, and Na [11,17,18,19,20,21]. A few years ago, we can specify, for example, the development of (Ba_1−y_Sr_y_)(Ti_1−x_Zr_x_)O_3_ (BSTZ) ceramics, which possess attractive electromechanical properties with a thickness coupling coefficient of 45% ${(k}_{t})$ for ultrasonic transducers [19]. In addition, there is some work referring to Hf-doped BaTiO_3_ (BHT), and these studies show that this system has attractive properties, e.g., a $k_{33}=57\%$ [22]. Therefore, the nature and concentration of the dopant are important criteria, considering their influences on BT piezoelectric performance against T_C_, which must not be too low to consider industrial development in accordance with targeted applications. Regarding BT doping with hafnium, it was shown that the material has attractive piezoelectric properties at doping rates of less than 15%, with an enhancement of approximately 5% and a T_C_ of ≈100 °C [22,23]. Moreover, an applicative purpose requires stable properties in terms of temperature. The hafnium doping rate must not induce an excessive decrease in the Curie temperature [15]. Thus, this work mainly focused on the synthesis and properties of BT doped with 5% hafnium, although some results are presented for other hafnium concentrations to show that the synthesis can be carried out with other rates of hafnium doping.

The conventional synthesis of BHT involves a solid-state reaction between titanium oxide, hafnium oxide, and barium carbonate. However, this method requires a high sintering temperature and can result in an inhomogeneous material with approximate stoichiometry [24]. Wet chemical syntheses, such as hydrothermal, coprecipitation, and sol–gel methods, make it possible to overcome these problems. Among these methods, the sol–gel process used to prepare BHT allows stable BHT sol to be obtained with a maximum concentration of 0.5 molar (M) due to the low hafnium and barium solubility. In addition, the current process uses strong organic complexes, such as acetylacetone or diethanolamine, which can lead to the formation of barium carbonates, resulting in the deterioration of ceramic properties [25,26].

In view of a subsequent energy-harvesting application [27], a composite sol–gel route must be used, consisting of a mixture of sol and powder, adapted from previous studies for PZT sols [28,29]. This process has already made it possible to obtain efficient thick films of adequate dimensions (lateral and thickness) typically via dip-coating, and in the long term, we wish to use it for the manufacture of bimorphs [26]. However, at first, this process requires a sol with specific features: a high concentration, adjustable viscosity, and time stability. To validate the first steps of this objective, we present the properties of bulk materials made from the BHT powder prepared from the sol, as well as feasibility tests of films made from the pure sol.

For this, we detail the synthesis of BHT sol from a mixture of Ba^2+^, Hf^4+^, and Ti^4+^ solutions with different Hf^4+^ doping rates of x = 0.05, 0.075, and 0.10, denoted as BHT5, BHT7.5, and BHT10, respectively. The reactivity of the precursor was adjusted to obtain a limpid solution of BHT. The solution rheology was studied to control parameters such as viscosity, particle size, and time stability.

Then, homogeneous thin films were deposited from this sol. The crystallization of the films during their heating treatment was monitored.

Finally, the previously mentioned sols were used to produce BHT powder, allowing the preparation of densified sintered pellets. These ceramics were poled, and their electromechanical and dielectric properties are presented to quantify the effectiveness of the process.

## 2. Materials and Methods

### 2.1. Pure Sol Preparation

**Figure 1:**
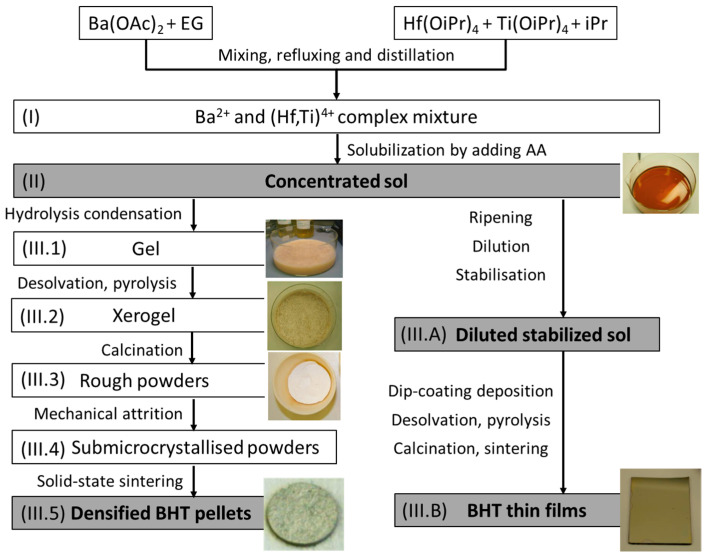
Steps and their corresponding pictures of BHT sol preparation for piezoelectric bulk ceramics and annealed thin films.

The sol–gel preparation principle has been previously used for the synthesis of PZT sol [28,29]. It was adapted for the synthesis of BHT sol, and the overall steps are schematized in Figure 1.

First, the precursor solution containing Ba^2+^ cations was realized by dissolving barium acetate (Ba(CH_3_COO)_2_; Sigma Aldrich 99%) at 0.5 M in hot ethylene glycol (EG) at 70 °C, below the saturation concentration.

Second, titanium and hafnium isopropoxide (Ti(OiPr)_4_, Merk ≥ 98% Hf(OiPr)_4_; Alpha Aesar, 99.9%) were dissolved in isopropanol (iPr) in a glove box under an inert atmosphere (Ar) and mixed vigorously for 1 h (h).

The two solutions were mixed and distilled thereafter at a temperature of up to 160 °C. This stage ensures the transalcoholysis of metallic ions, i.e., removing isopropanol and isopropoxide molecules to create glycolate ligands (step I in Figure 1). Distillation monitoring was carried out using Fourier-transform infrared (FTIR) spectroscopy with a PerkinElmer Spectrum Two spectrometer (PerkinElmer, Waltham, MA, USA).

Glacial acetic acid (AA) was added at 100 °C to solubilize the complex mixture and prevent precipitation during cooling. A yellowish 0.9 M concentrated sol was finally obtained (step II in Figure 1), which could be used later to elaborate both bulk ceramics (left branch of Figure 1) or thin films (right branch of Figure 1).

### 2.2. Thin Film Elaboration

We first confirmed that homogeneous thin films can be processed with a binder alone. Starting with the concentrated sol elaborated in step II (Figure 1), the solution was left to mature for 20 days and then diluted with ethylene glycol to obtain a stable 0.5 M solution (step III.A, right branch in Figure 1). The ripening of sols was studied via rheology with a rotational viscosimeter (Haake Rheostress 1,Thermo Fischer Scientific, Waltham, MA, USA) Colloid sizes were evaluated using dynamic light scattering (Malvern Zetasizer nona-ZS apparatus, Malvern, Worcestershire, United Kingdom).

Prior to deposition, the substrates (Si/Pt) were washed using deionized water. The nonionic surfactant Triton X-100 (TX, 1% wt.) was added to the 0.5 M stabilized sol. The film deposition was carried out via dip-coating at room temperature with pilling speeds of up to 10 cm/min. The film was first subjected to a thermal treatment of up to 600 °C on a heat plate and finally annealed in an RTA oven at up to 900 °C. The process was controlled using FTIR spectroscopy (Nicolet Magna IR 550 series II, Thermo Fischer Scientific, Waltham, MA, USA) every 50 °C to finally obtain a thin BHT film whose composition was verified via Rutherford backscattering spectrometry (RBS, CEA DAM, Bruyères le Châtel, Isles-de-France, France) (step III.B, right branch in Figure 1).

### 2.3. Ceramic Elaboration

As for the thin film, the powder elaboration started from the concentrated sol in step II (Figure 1). Water (20% in weight (wt)) was added to the sol to initiate the hydrolysis/condensation reaction and left under continuous stirring until gel formation (step III.1, left branch in Figure 1). The gel was dried at 70 °C for one day to ensure the evaporation of the solvents (step III.2, left branch in Figure 1).

To determine the thermal treatment conditions of the sol, thermal decomposition analysis was performed via thermogravimetric–differential thermal analysis (TGA/DTA) under an air atmosphere (Netzsch STA 409, Netzsch Gmbh , Selb, Bavaria, Germany), and the xerogel crystallization was monitored using X-ray diffraction (XRD) every 100 °C (XPERT PRO diffractometer, Cu wavelength λ= 1.54 Å, Malvern Panalytical Ltd., Malvern, Worcestershire, United Kingdom). The prepared white powder was ground in a mortar to obtain a fine powder, and its chemical composition was analyzed using inductively coupled plasma–mass spectrometry (ICPMS, NexION 350XX PerkinElmer, Perkin Elmer, Waltham, MA, USA) (step III.3, left branch in Figure 1).

To obtain a submicrocrystalline powder, mechanical attrition (Netzsch PE07, Netzsch Gmbh , Selb, Bavaria, Germany) was performed on the crystallized BHT powders at room temperature (~20 °C, at 1500 rounds per minute) (step III.4, left branch in Figure 1): 75 g of BHT powder was mixed with 150 g of ethanol and 1.6 kg of 0.5 mm diameter zirconium beads for a time ranging from 5 to 20 min (min). The granulometry of the obtained powder was controlled using dynamic light scattering (Malvern Zetasizer nonaZS, Malvern Panalytical Ltd., Malvern, Worcestershire, United Kingdom), scanning electron microscopy (SEM, Zeiss LEO 435, Zeiss AG., Oberkochen, Germany), and transmission electron microscopy (TEM, JEOL 2100F, JEOL Ltd., Akishima, Tokyo, Japan).

The sintering temperature was determined via dilatometry analysis (SETARAM Setsys Evolution dilatometer, KEP Technologies Inc., Austin, TX, USA) in a temperature range from room temperature to 1500 °C with a heating rate of 1 °C/min. The BHT powders were uniaxially pressed at 20 Megapascals (MPa), using polyvinyl alcohol as a binder, to form 16 mm diameter and 1 mm thick pellets. The powders were sintered using different times and temperatures to obtain bulk ceramics (step III.5, left branch in Figure 1).

The crystallographic analysis of the bulk ceramics was performed using X-ray diffraction (D8 Brucker Advance diffractometer Cu wavelength λ = 1.54 Å, Bruker Corp., Billerica, MA, USA) and the sample microstructures were observed using scanning electron microscopy (Zeiss LEO 440, JEOL Ltd., Akishima, Tokyo, Japan).

Silver electrodes were deposited on each side of the pellets via sputtering to pole the pellets using field cooling at a constant voltage (~400 V/mm) in an oil bath at 140 °C.

The electromechanical parameters of the pellets were deduced from the measurements of the complex electrical impedance as a function of the frequency around the fundamental thickness-mode resonance in air using an HP4395 spectrum analyzer and its impedance test kit (Agilent Technologies Inc., Santa Clara, CA, USA). An equivalent electrical circuit model was used to simulate the behavior of the electrical impedance of the samples as a function of frequency for the thickness mode. The model retained was the Krimholtz–Leedom–Matthaei (KLM) scheme [30], where dielectric and mechanical losses were introduced [31]. This one-dimensional (1D) model allows the separation of the network of the acoustic and electrical ports of a piezoelectric element. A matrix formula was implemented for the numerical calculation of the structure [32].

From the measurement of the anti-resonant frequency ($f_{a}$, at the maximum value of the impedance), the longitudinal wave velocity ($v_{l}$) was deduced with the following relation:

(1) $$v_{l}=2\cdot t\cdot \;f_{a}$$

where t is the thickness of the pellets. The elastic parameter at a constant electrical displacement ($C_{33}^{D}$) was calculated according to:

(2) $$C_{33}^{D}=v_{l}^{2}\cdot \rho$$

where *ρ* is the density of the pellet. With a fitting process for the complex experimental electrical impedance, the effective thickness coupling factor ($k_{t}^{}$) and the dielectric constant at a constant strain $\varepsilon_{33}^{S}/\varepsilon_{0}$ were deduced. Finally, according to the thickness coupling factor formula, the piezoelectric coefficient *e*_33_ was obtained:

(3) $$e_{33}^{}=k_{t}^{}\cdot \sqrt{C_{33}^{D}\cdot \varepsilon_{33}^{S}}$$

This experimental setup was also used to determine the capacitance at a frequency of 1 kHz and as a function of temperature to determine the Curie temperature of the samples.

## 3. Results

### 3.1. Pure Sol Characterization, Stabilization, and Thin Film Elaboration

#### 3.1.1. Sol Characterizations

Ethylene glycol was chosen as the solvent to prepare a highly concentrated sol with controlled colloid sizes. This solvent can act as a stabilizing agent and increase the viscosity of the solution [28]. Isopropanol is mainly used to prevent the hydrolysis of titanium and hafnium with ethylene glycol, creating a screening effect between precursors [33,34]. When barium solution was slowly added to the latter solution of titanium/hafnium, no immediate gel formation occurred.

**Figure 2:**
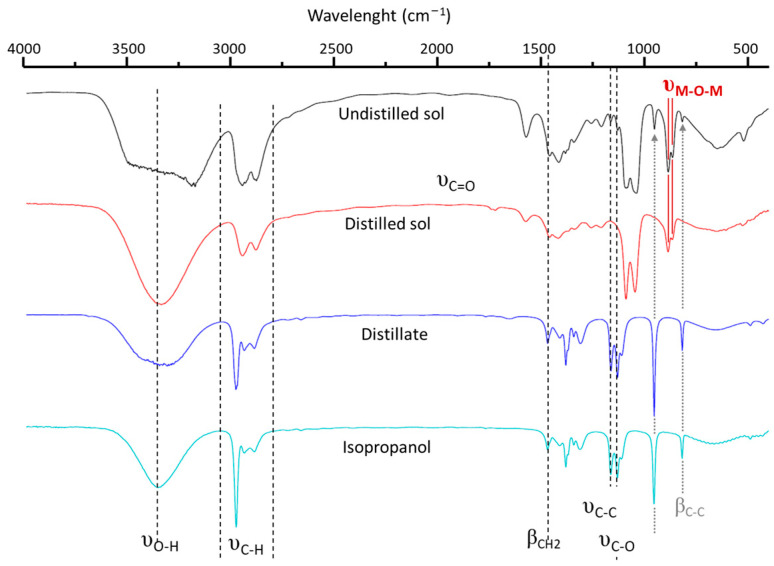
FTIR spectra of BHT5 sol before (black solid line) and after distillation (red solid line) process in comparison with distillate (dark blue solid line) and used isopropanol solvent (blue solid line).

To study the various processes occurring during distillation, the distillate and distilled BHT5 sol were analyzed via FTIR. The spectra obtained are depicted in Figure 2 and compared with those of isopropanol and undistilled sol.

The distillate and pure isopropanol spectra (Figure 2) present similar peaks centered at 3330 cm^−1^ (υ_O−H_ stretching band), 2970–2880 cm^−1^ (υ_C−H_ stretching band), 1390 cm^−1^ (υ_C−H3_ splitting mode), 1130–810 cm^−1^ (C-O bond), and 950 cm^−1^ (C-CH_3_ stretching mode), confirming that the distillate was mainly composed of isopropanol [33]. Moreover, the weight of alcohol eliminated via distillation represented approximately 90% wt of the total isopropanol moiety of the initial solution (solvent and isopropoxide). This indicates that isopropoxide ligands were replaced in the vicinity of metallic ions.

The common peaks in the spectra of the distilled and undistilled sols, centered at 3330 cm^−1^ (υ_C−H_ stretching band), 2950–2880 cm^−1^ and the 1580 cm^−1^ band (CO_2_- symmetric and asymmetric stretching), 1450–1210 cm^−1^ (CH_2_ twisting), 1080 and 1040 cm^−1^ (C-C and C-O bond), and 880 cm^−1^ (CH_2_ rocking), confirm the presence of ethylene glycol and acetate in the sol [34]. The undistilled sol spectrum shows additional peaks at 950 and 820 cm^−1^ corresponding to the CH_3_-C-CH_3_ and asymmetric C-O stretching vibrations of isopropanol.

Consequently, FTIR analysis confirmed the elimination of isopropanol and the ligand exchange of metallic ions.

#### 3.1.2. Ripening and Stabilization of the Sol

**Figure 3:**
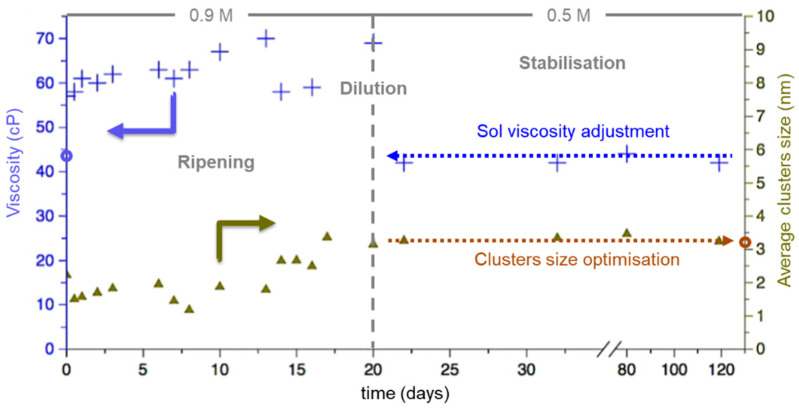
Time dependence of the viscosity (blue crosses) and average cluster size (green triangles) of BHT5 sol with an initial concentration of 0.9 M and after dilution at 0.5 M.

During the ripening of the sol, the BHT5 cluster sizes grew and impacted the sol viscosity. Figure 3 shows the time evolution of the particle sizes and sol rheology over 120 days.

In the first 20 days, the viscosity of the prepared 0.9 M BHT5 sol showed an increase from 50 centiPoise (cP) to nearly 70 cP. The DLS analysis shows that this rheological behavior can be directly correlated with the growth of clusters up to 3 nanometers (nm).

After 20 days of ripening, the cluster growth process was stopped by diluting the sol in ethylene glycol to obtain a 0.5 M concentration. This behavior is depicted in Figure 3. Between 20 and 100 days, dilution decreased the viscosity from 70 cP to 45 cP, and the cluster size remained constant at ~3 nm.

This shows that with this dilution step, the granulometric and rheological parameters of solutions can be controlled, and the sol is stable at room temperature over 3 months.

#### 3.1.3. Thin Film Elaboration

**Figure 4:**
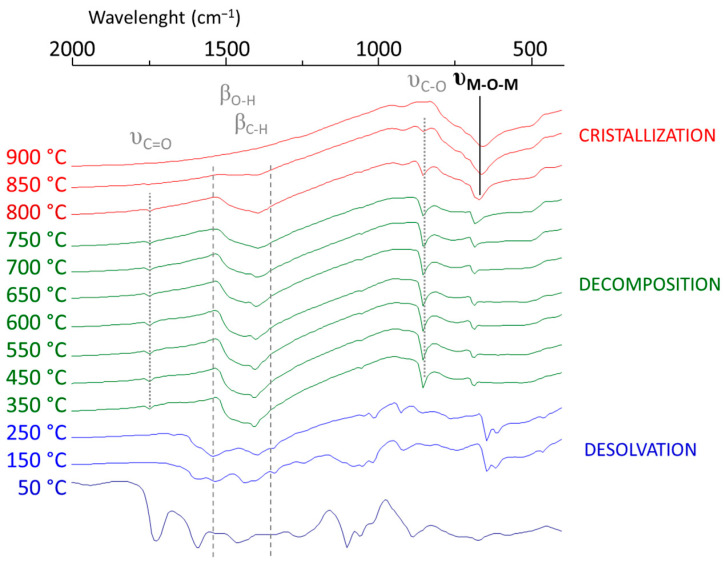
FTIR spectra of BHT5 sol during the crystallization process with annealing between 50 and 900 °C.

The stable BHT sol obtained was used to process homogenous thin films. The layer was submitted to thermal treatment, and the film formation was monitored using FTIR spectroscopy (Figure 4).

Therefore, the overall sequence consisted of the following steps:

1. Up to 250 °C, a desolvation step to obtain the dried sample was performed, depicted in the disappearance of the 1710, 1100, and 1000 cm^−1^ bands of ethylene glycol and acetic acid and the peaks at 1550 and 1400 cm^−1^ corresponding to the symmetric and asymmetric C-O stretching bands of carboxylic acid groups.
2. The decomposition of the last organic parts occurred during 2 calcination steps (450 °C for 10 min and then 600 °C for 20 min.) The remaining acetate started to decompose, creating carbonate, which appeared at 1750 and 1410 cm^−1^ in the spectra.
3. The multilayer film was crystallized with the complete removal of carbonates at 900 °C for 10 min. The crystallization of BHT films was confirmed by the disappearance of the characteristic IR bands of carbonate groups, along with the appearance of metal–oxide–metal (M-O-M) bands at 660 cm^−1^.

Thereafter, the deposition crystallization and thermal annealing of films with additional doping rates of 7.5 and 10% in hafnium (BHT7.5; BHT10) were performed. Their compositions were evaluated using RBS analysis, and the results are reported in Table 1 and discussed thereafter. An image of the thin film realized with the BHT5 sol is presented in Figure 1 (step III.B, right branch in Figure 1).

The different characterizations confirm that a highly concentrated and time-stable sol of BHT can be synthesized with different hafnium doping rates between 5 and 10%. Its viscosity can be easily adjusted to produce thin films that, after annealing, have similar compositions to the desired film, regardless of the hafnium doping rate between 5 and 10%.

### 3.2. Powders and Pellet Elaborations from Pure Sol

#### 3.2.1. Xerogel Crystallization

**Figure 5:**
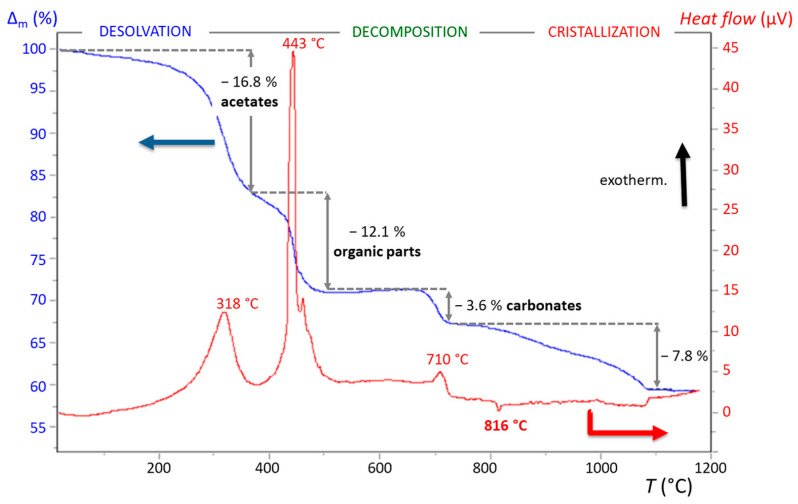
TGA (blue line)/TD (red line) thermal analysis with annotated compounds that decomposed during the annealing of BHT5 sol (∆m: relative mass variation).

After the hydrolysis/condensation process, thermogravimetric–DTA analysis was performed on a dried gel to obtain a BHT5 microcrystallized powder (Figure 5).

The thermal analysis of the BHT5 sol depicts a small weight variation from room temperature to 200 °C due to the evaporation of solvents and residual water and then clearly exhibits three successive exothermic peaks accompanied by significant weight loss corresponding to the decomposition reactions of acetates (318 °C), organic compounds (443 °C), and carbonates (710 °C). The endothermic peak at 816 °C, regardless of the dopant content, is related to the formation of perovskite phases.

**Figure 6:**
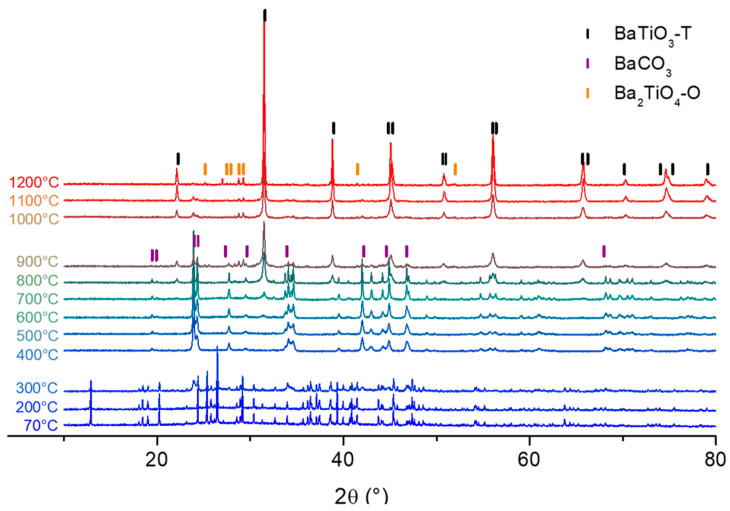
XRD patterns as a function of temperature for BHT5 dried gel with indexations of tetragonal BaTiO_3_ (PDF#01-74-1956), BaCO_3_ (Crystallography Open Data, C.O.D. 1000033) and Ba_2_TiO_4_ (C.O.D. 2310344) phases.

To follow the crystallization process of the perovskite phase, XRD analysis with respect to temperature was performed (Figure 6).

Between 70 and 300 °C, the process began with the desolvation of the gel as acetate was released (Figure 6). Between 400 and 900 °C, pyrolysis of the remaining organics occurred. This pyrolysis was accompanied by the appearance of BaCO_3_, as shown by the appearance of 2θ = 24, 34, and 42° peaks in the diffraction pattern (Figure 6). The disappearance of those peaks indicates the decomposition of carbonate between 1000 and 1200 °C (Figure 5 and Figure 6) and those leading to the formation of a tetragonal BaTiO_3_-like phase (BaTiO3-T, PDF#01-74-1956), which started to appear at approximately 800 °C and was contaminated with a small amount of the Ba_2_TiO_4_ orthorhombic phase (Ba_2_TiO_4_-O).

The overall process leading to the formation of the Ba_2_TiO_4_ phase can be explained by the decomposition of acetate up to 450 °C, leading to the formation of barium carbonate BaCO_3_ [35]. At higher temperatures, BaCO_3_ undergoes decarbonation, resulting in BaO. This latter can react with BaTiO_3_ to form the Ba_2_TiO_4_ phase [36].

Consequently, to remove whole organic compounds and prevent the formation of auxiliary phases, pyrolysis at 400 °C for 4 h on dried and ground gel powder was carried out, followed by crystallization at 900 °C in a furnace, ensuring the formation of single-phase perovskite. This process was employed to obtain powders with different hafnium doping rates of 5, 7.5, and 10%.

The stoichiometry of the powder was verified with ICPMS on BHT5, 7.5, and 10% and compared with RBS analysis carried out on thin films deposited from diluted sol. The results are reported in Table 1.

#### 3.2.2. Mechanical Attrition

A mechanical attrition process was applied to initially crystallized 1 to 5 µm size BHT powders. The combination of wet-milling and fine-grinding processes allowed a reduction in the average grain size by a factor of 5. The microstructural changes in the attrited BHT particles were observed using TEM analysis.

**Figure 7:**
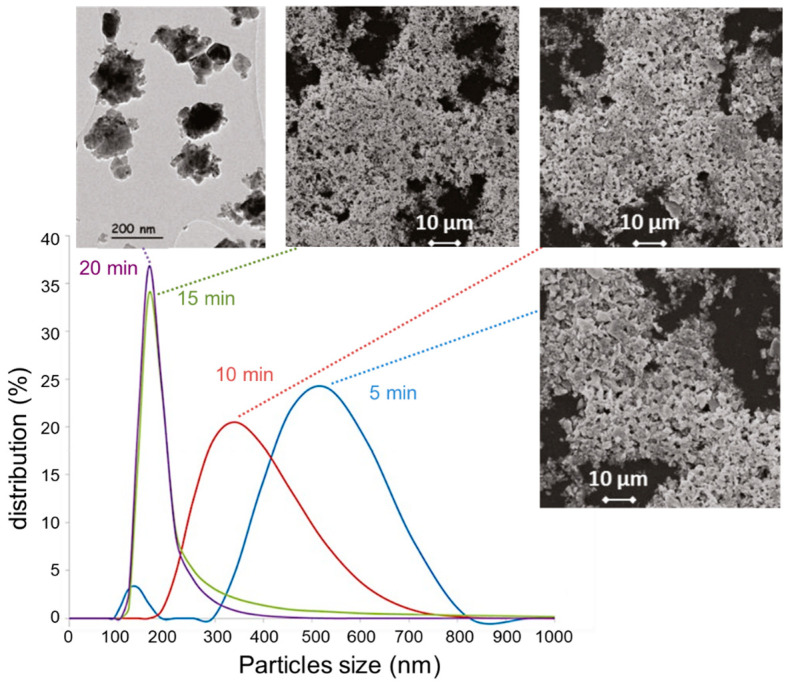
Average particle diameter of crystallized BHT5 powders after the mechanical attrition process at different times.

After mechanical attrition for 5 min or 10 min, the average particle diameter was significantly reduced to approximately 515 ± 120 nm or 340 ± 100 nm, respectively (Figure 7).

The decrease in the particle sizes, as well as their size distributions, was achieved after 15 min of attrition and confirmed after 20 min. Consequently, the BHT5, 7.5, or 10 attrited powders, studied afterward, were realized with attrition for 20 min.

#### 3.2.3. Sintering Conditions of the Pellets

For the non-attrited BHT powders, sintering occurred at 1460 °C for BHT5 and lowered at approximately 1350 °C and 1400 °C for BHT7.5 and BHT10, respectively, close to the BaTiO_3_ reference (1300 °C). The ending point was between 1410 °C and 1550 °C with a low shrinking rate of approximately 10%. Thus, all compositions presented a close range of sintering temperatures between 1400 and 1500 °C.

As the sintering temperature presented a slight variation upon hafnium doping, a dilatometric study was also carried out on the intermediate composition (BHT7.5) powder. The deduced sintering temperatures were lower than 1350 °C, and the shrinkage was larger (~35%). This demonstrates that the reduction in the grain size of the powder via attrition allowed a better densification of the ceramic while applying a lower sintering temperature.

**Figure 8:**
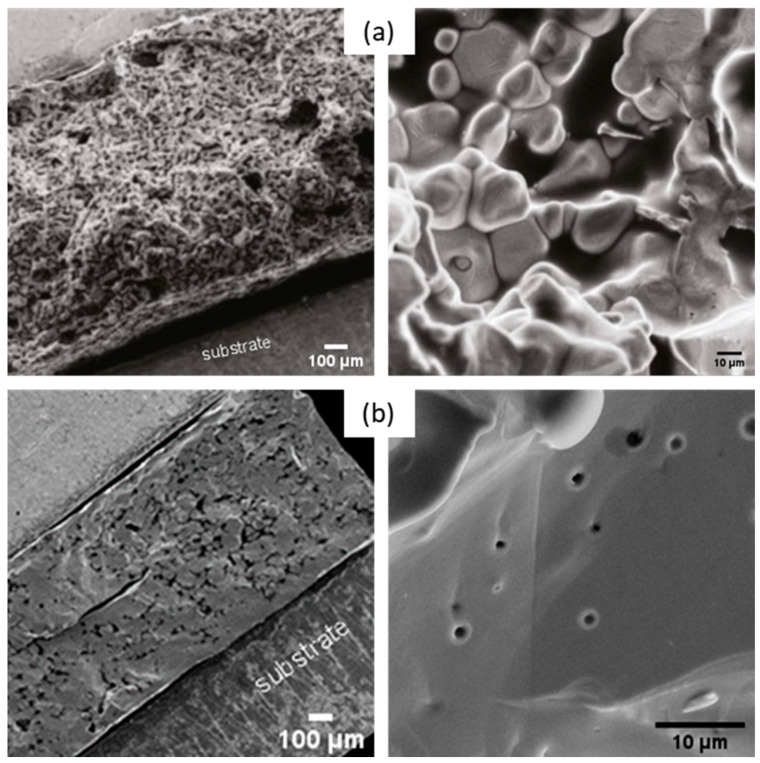
SEM images of non-attrited (**a**) and attrited (**b**) BHT5 sintered at 1500 °C for 4 h.

The influence of attrition on sintered pellets was investigated via SEM. Figure 8 displays the micrographs of the pellets made of non-attrited (Figure 8a) and attrited BHT5 powders (Figure 8b) sintered at a sintering temperature (T_s_) corresponding to the endpoint of the dilatometric measurements at T_s_ = 1500 °C, with a typical dwelling time (4 h) used for this material [37].

Non-attrited BHT5, sintered at 1500 °C (Figure 8a), shows a morphology formed by grains linked together by grain joints. However, the picture also shows an important residual porosity. Despite a sufficient temperature to initiate the sintering process, the coarse morphology of the powder did not allow significant densification of the pellet after sintering. As exhibited in Figure 8b, the attrition of the powder and the grain size reduction induced better densification of the material, as the latter shows a uniform morphology, although some pores and cracks are still present.

**Figure 9:**
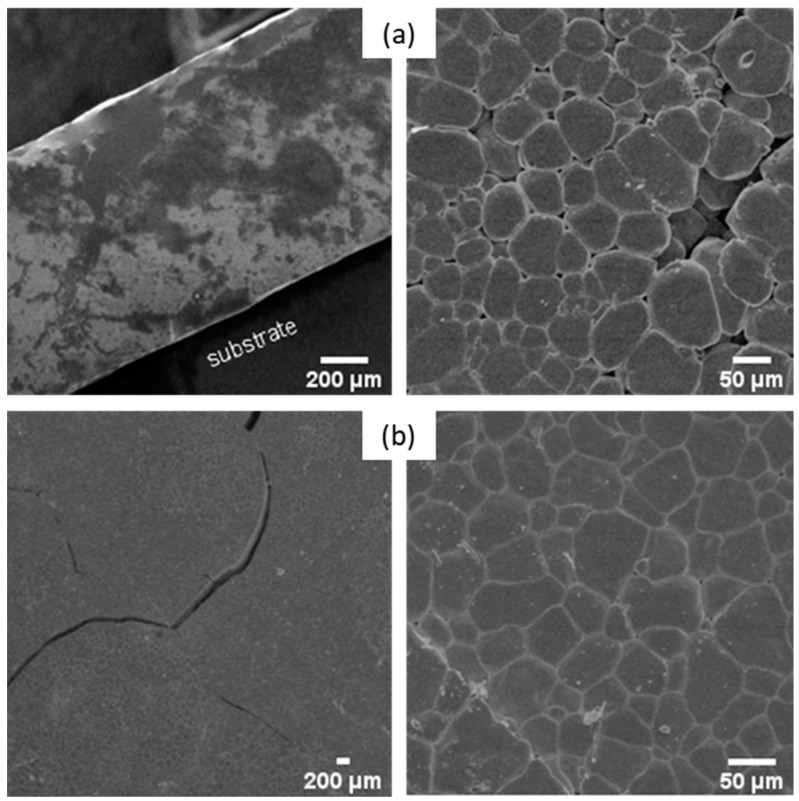
SEM images of attrited BHT5 morphology sintered for 6 h (**a**) and 10 h (**b**) at 1500 °C.

To eliminate the residual porosity in the attached pellets, the sintering time duration was increased from 4 h to 10 h at 1500 °C. The morphologies of these samples were observed using SEM and are presented in Figure 9.

Although the porosity of the samples seems to decrease with a longer sintering time, the samples show cracks and defects. A sintering time of 6 h is a good trade-off between the reduction in porosity and the formation of cracks.

**Figure 10:**
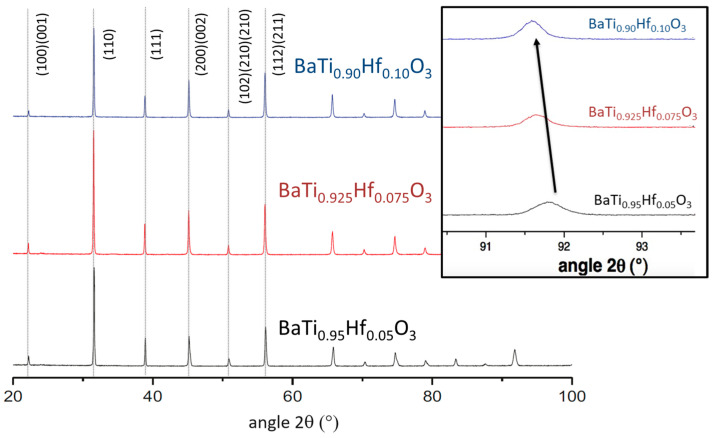
X-ray patterns at room temperature of BHT5, 7.5, and 10 pellets (inset: magnification of approximately 92°).

The X-ray diffraction patterns at room temperature of the sintered powders (Figure 10) confirm the formation of single-phase perovskite in comparison with tetragonal BaTiO_3_ (BaTiO_3_-T, PDF#01-74-1956), corresponding to the desired perovskite single phase [38,39].

We can notice an evolution of the peak centered at 2θ = 91.7 ° (inset of Figure 10) with a shift to 2θ = 91.5 ° from x = 0.05 to x = 0.10. This variation is due to the insertion of Hf in the lattice, as this induces a distortion of the crystalline structure and hence confirms the absence of phase segregation between BaHfO_3_ and BaTiO_3_.

#### 3.2.4. Dielectric Properties of BHT5 Ceramic

**Figure 11:**
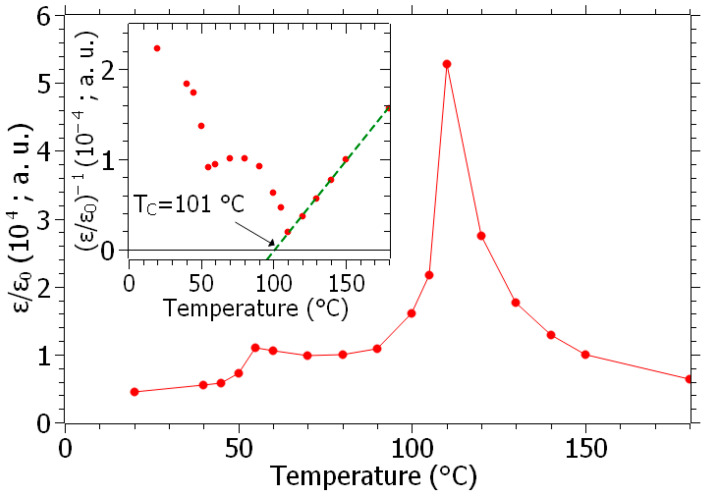
Temperature dependence of the relative permittivity (red) of the BHT5-attached pellet (inset: inverse of permittivity (red dots), and linear interpolation (green dashed line)).

A 13 mm diameter and 1 mm thick pellet, sintered at 1500 °C for 4 h, was covered with 150 nm of Al deposited via magnetron sputtering on both sides of the ceramic. The temperature dependence of the capacitance of the attrited BHT5 pellet is displayed in Figure 11.

A material’s capacitance (*C*) is directly linked to its relative permittivity ($\varepsilon_{r}$) as [40]:

(4) $$\varepsilon_{0}\cdot \varepsilon_{r}=\frac{C\cdot t}{A}$$

where $\varepsilon_{0}$ is the vacuum permittivity, and t and *A* are the sample thickness and area, respectively. The capacitance (Figure 11) shows two peaks located at 55 and 110 °C, corresponding to the enhancement of the permittivity near the orthorhombic–tetragonal and tetragonal–cubic transition temperatures. Above the Curie temperature ($T_{C}$), the temperature (*T*) dependence of the permittivity is usually described with the Curie–Weiss law [41]:

(5) $$\varepsilon_{r}\propto {\left(T-T_{c}\right)}^{-1}$$

Thus, reciprocal permittivity $S\propto \varepsilon_{r}^{-1}$ shows a linear dependence vs. temperature, as depicted in the inset of Figure 11. By extrapolation to S→0, the Curie temperature is determined at $T_{c}$ = 101 °C, in accordance with the literature [18,23,38]. For our targeted application (energy harvesters), this value is completely satisfactory and not handicapping for devices operating at room temperature and without significant heating.

#### 3.2.5. Electromechanical Properties of BHT Ceramics

First, the properties of BHT5, 7.5, and 10 pellets made from attrited powders with a common sintering temperature of 1400 °C for 4 h were investigated. All investigated BHT ceramics sintered in these conditions showed low relative densities (55%<d<75%). The three samples delivered comparable electromechanical properties with a thickness coupling coefficient ranging from 31% to 34%, a dielectric constant at constant strain ${(\varepsilon }_{33}^{s}{/\varepsilon }_{0}^{})$ between 660 (BHT5) and 790 (BHT10), and high mechanical losses (of over 15%), mainly due to high porosity contents [42]

**Figure 12:**
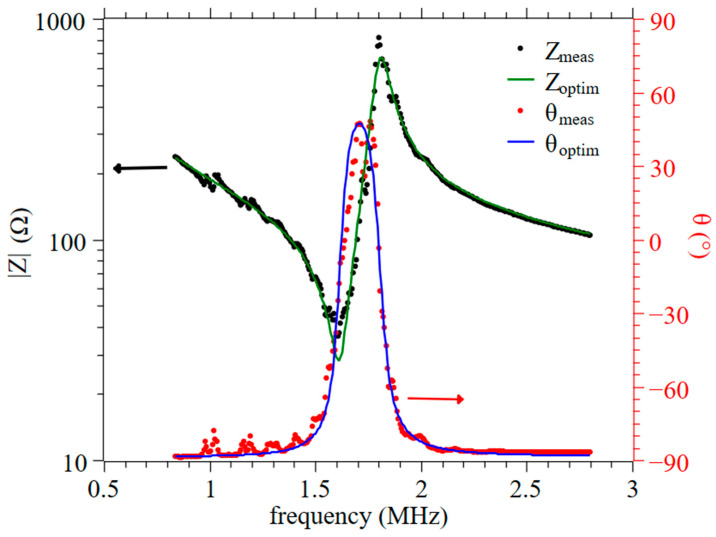
Impedance (Z, black dots) and phase (θ, red dots) measured (meas) in poled attrited BHT5 sintered at 1500 °C for 6 h and their respective optimized (optim) theoretical curves (green and blue lines) using KLM model.

The second series of characterizations then focused on the BHT5 samples with attrited and non-attrited powders. Samples were obtained with different sintering times or temperatures. The impacts of these processing conditions on the electromechanical properties are summarized in Table 2. Figure 12 also provides an example of the measured and optimized impedance curve for the attrited BHT5 ceramic sintered at 1500 °C for 6 h.

The attrition milling process clearly had a significant impact on the improvement in the density (from 60% to 70% for the relative density) with the same sintering conditions (1500 °C/4 h). This densification was accompanied by the enhancement of $\varepsilon_{33}^{S}/\varepsilon_{0}$, $e_{33}$, and $C_{33}^{D}$.

When the sintering temperature increased to 1500 °C with 4 or 6 h of dwelling time, the final densities, dielectric constant at constant strain, and piezoelectric coefficient remained almost equal to $d=72\pm 2\%,\;700\;and\;12\;C\cdot m^{-2},\;respectively$. The first difference occurred in the mechanical constant, $C_{33}^{D},$ which slightly decreased for the following conditions: 1500 °C/6 h. This led to a small increase in the thickness coupling factor (from 45 to 47%), but these variations can be considered within measurement errors. Moreover, the mechanical losses significantly decreased for the last sintering condition (4%), which went hand in hand with better densification. To conclude, the $k_{t}$ value was similar to that of conventional PZT [8,43].

## 4. Conclusions

Sol–gel synthesis was established for BHT with appropriate characteristics for use in film processing. Distillation allowed sol to be prepared at high concentrations, nearly up to 1 M, with nanometric colloids that enhanced viscosity. Dilution not only permitted the stabilization of the sols but also set the desired characteristics. The prepared sols were verified to be precursors of a unique perovskite phase, and the solid solutions of BaHfO_3_ and bulk materials showed very good electromechanical properties ($k_{t}=$47%) comparable to standard PZT for BHT5 with optimized sintering conditions. Moreover, we demonstrated the feasibility of a thin film fabricated via spin-coating with this concentrated sol and whose composition is very close to the desired composition. From the perspective of being able to develop lead-free thick films needed in vibrational piezoelectric energy-harvesting devices, the optimization of the composite sol–gel (mixture of the sol and the powder) route and their deposition conditions are under investigation. Optimizing these processing conditions will enable the development of bimorph cantilevers, whose electromechanical behavior can be modeled and optimized using finite element models and characterized with electrical measurements (impedance as a function of frequency) using an impedance analyzer and vibration measurements (output harvested power as a function of acceleration) using a dedicated test bench.

## Figures and Tables

**Table 1:** Quantitative analysis (RBS and ICPMS) of the three BHT sols and corresponding thin films.

Sample	Theoretical Composition	BHT Thin Film (RBS)	BHT Sol (ICPMS)
BHT5	Ba_1_Ti_0.950_Hf_0.050_O_3_	Ba_0.990_Ti_0.969_Hf_0.038_O_3.003_	Ba_1.100_Ti_0.888_Hf_0.045_O_2.967_
BHT7.5	Ba_1_Ti_0.925_Hf_0.075_O_3_	Ba_1.002_Ti_0.917_Hf_0.081_O_2.999_	Ba_1.166_Ti_0.832_Hf_0.056_O_2.945_
BHT10	Ba_1_Ti_0.900_Hf_0.100_O_3_	Ba_1.040_Ti_0.867_Hf_0.106_O_2.987_	Ba_1.032_Ti_0.883_Hf_0.095_O_2.989_

**Table 2:** Properties of BHT5 ceramics prepared under various conditions (powder and sintering).

Sintering Conditions		$\rho \left(g\cdot cm^{-3}\right)/d$ (%)	$C_{33}^{D}$ (GPa)	$\varepsilon_{33}^{S}/\varepsilon_{0}$	$\delta_{m}$ (%)	$e_{33}$ (C/m^2^)	$k_{t}$ (%)
T (°C)/Times (h)	Powder						
1500/4	N.A.	3800/60	42	570	17	5	34
1400/4	A.	4620/74	79	660	20	7	31
1500/4	A.	4420/70	111	695	11	12	45
1500/6	A.	4520/73	101	700	4	12	47

A.: attrited; N.A.: non-attrited; ρ: density; $d=\rho /\rho_{0}$: relative density, $\rho_{0}$ = 6.22 g/cm^3^; $e_{33}$: piezoelectric coefficient; $C_{33}^{D}$: elastic constant at constant electrical displacement; $\delta_{m}$: mechanical losses; $\varepsilon_{33}^{S}/\varepsilon_{0}$: dielectric permittivity at constant strain; $k_{t}$: thickness coupling factor.

## Data Availability

Data available on request from the authors.

## References

[1] Hoffman J., Hong X., Ahn C.H. (2011). Device performance of ferroelectric/correlated oxide heterostructures for non-volatile memory applications. Nanotechnology.

[2] Sheng S., Ong C.K. (2012). Coupled microstrip line microwave phase shifter using ferroelectric thin film varactors. J. Appl. Phys..

[3] Yin S., Niu G., Vilquin B., Gautier B., Le Rhun G., Defay E., Robach Y. (2012). Epitaxial growth and electrical measurement of single crystalline Pb(Zr_0.52_Ti_0.48_)O_3_ thin film on Si(001) for micro-electromechanical systems. Thin Solid Films.

[4] Guillon O., Chang J., Schaab S., Kang S.-J.L. (2012). Capacitance Enhancement of Doped Barium Titanate Dielectrics and Multilayer Ceramic Capacitors by a Post-Sintering Thermo-Mechanical Treatment. J. Am. Ceram. Soc..

[5] Yang X., Su X., Shen M., Zheng F., Xin Y., Zhang L., Hua M., Chen Y., Harris V.G. (2012). Enhancement of Photocurrent in Ferroelectric Films Via the Incorporation of Narrow Bandgap Nanoparticles. Adv. Mater..

[6] Zhou Q.F., Chan H.L.W., Choy C.L. (2000). PZT ceramic/ceramic 0–3 nanocomposite films for ultrasonic transducer applications. Thin Solid Films.

[7] Lukacs M., Sayer M., Foster S. (2000). Single element high frequency (<50 MHz) PZT sol gel composite ultrasound transducers. IEEE Trans. Ultrason. Ferroelectr. Freq. Control.

[8] Panda P.K., Sahoo B. (2015). PZT to Lead Free Piezo Ceramics: A Review. Ferroelectrics.

[9] The European Parliamant, the Concil of European Union (2011). Directive 2011/65/EU of the European Parliament and of the Council of 8 June 2011 on the restriction of the use of certain hazardous substances in electrical and electronic equipment. Off. J. Eur. Union.

[10] Hoang T., Poulin-Vittrant G., Ferin G., Levassort F., Bantignies C., Nguyen-Dinh C., Bavencoffe M. (2018). Parametric study of a thin piezoelectric cantilever for energy harvesting applications. Adv. Appl. Ceram..

[11] Rödel J., Jo W., Seifert K.T.P., Anton E.M., Granzow T., Damjanovic D. (2009). Perspective on the development of lead-free piezoceramics. J. Am. Ceram. Soc..

[12] Devonshire A.F. (1949). XCVI. Theory of barium titanate. Lond. Edinb. Dublin Philos. Mag. J. Sci..

[13] Devonshire A.F. (1951). CIX. Theory of barium titanate—Part II. Lond. Edinb. Dublin Philos. Mag. J. Sci..

[14] von Hippel A. (1950). Ferroelectricity, Domain Structure, and Phase Transitions of Barium Titanate. Rev. Mod. Phys..

[15] Kalyani A.K., Brajesh K., Senyshyn A., Ranjan R. (2014). Orthorhombic-tetragonal phase coexistence and enhanced piezo-response at room temperature in Zr, Sn, and Hf modified BaTiO_3_. Appl. Phys. Lett..

[16] Zhu X.N., Zhang W., Chen X.M. (2013). Enhanced dielectric and ferroelectric characteristics in Ca-modified BaTiO_3_ ceramics. AIP Adv..

[17] Dixit A., Majumder S.B., Savvinov A., Katiyar R.S., Guo R., Bhalla A.S. (2002). Investigations on the sol–gel-derived barium zirconium titanate thin films. Mater. Lett..

[18] Chen J., Fu C., Cai W., Chen G., Ran S. (2012). Microstructures, dielectric and ferroelectric properties of BaHf_x_Ti_1−x_O_3_ ceramics. J. Alloys Compd..

[19] Lee S.T.F., Lam K.H., Zhang X.M., Chan H.L.W. (2011). High-frequency ultrasonic transducer based on lead-free BSZT piezoceramics. Ultrasonics.

[20] Yan X., Lam K.H., Li X., Chen R., Ren W., Ren X., Zhou Q., Shung K.K. (2013). Correspondence: Lead-free intravascular ultrasound transducer using BZT-50BCT ceramics. IEEE Trans. Ultrason. Ferroelectr. Freq. Control.

[21] Sasaki A., Chiba T., Mamiya Y., Otsuki E. (1999). Dielectric and Piezoelectric Properties of (Bi_0.5_Na_0.5_)TiO_3_–(Bi_0.5_K_0.5_)TiO_3_ Systems. Jpn. J. Appl. Phys..

[22] Tian H.Y., Wang Y., Miao J., Chan H.L.W., Choy C.L. (2007). Preparation and characterization of hafnium doped barium titanate ceramics. J. Alloys Compd..

[23] Payne W.H., Tennery V.J. (1965). Dielectric and Structural Investigations of the System BaTiO_3_-BaHfO_3_. J. Am. Ceram. Soc..

[24] Vijatović M.M., Bobić J.D., Stojanović B.D. (2008). History and challenges of barium titanate: Part I. Sci. Sinter..

[25] del Carmen Blanco López M., Fourlaris G., Rand B., Riley F.L. (1999). Characterization of Barium Titanate Powders: Barium Carbonate Identification. J. Am. Ceram. Soc..

[26] Bardaine A., Boy P., Belleville P., Acher O., Levassort F. (2008). Influence of powder preparation process on piezoelectric properties of PZT sol-gel composite thick films. J. Solgel Sci. Technol..

[27] Ferin G., Hoang T., Bantignies C., Le Khanh H., Flesch E., Nguyen-Dinh A. Powering autonomous wireless sensors with miniaturized piezoelectric based energy harvesting devices for NDT applications. Proceedings of the 2015 IEEE International Ultrasonics Symposium (IUS).

[28] Belleville P., Bigarre J., Boy P., Montouillout Y. (2007). Stable PZT sol for preparing reproducible high-permittivity perovskite-based thin films. J. Solgel Sci. Technol..

[29] Bardaine A., Boy P., Belleville P., Acher O., Levassort F. (2008). Improvement of composite sol–gel process for manufacturing 40 μm piezoelectric thick films. J. Eur. Ceram. Soc..

[30] Krimholtz R., Leedom D.A., Matthaei G.L. (1970). New equivalent circuits for elementary piezoelectric transducers. Electron. Lett..

[31] Lethiecq M., Patat F., Pourcelot L., Tran-Huu-Hue L.P. (1993). Measurement of losses in five piezoelectric ceramics between 2 and 50 MHz. IEEE Trans. Ultrason. Ferroelectr. Freq. Control.

[32] van Kervel S.J.H., Thijssen J.M. (1983). A calculation scheme for the optimum design of ultrasonic transducers. Ultrasonics.

[33] Doeuff S., Henry M., Sanchez C., Livage J. (1987). Hydrolysis of titanium alkoxides: Modification of the molecular precursor by acetic acid. J. Non Cryst. Solids.

[34] Guglielmi M., Carturan G. (1988). Precursors for sol-gel preparations. J. Non Cryst. Solids.

[35] Hwang U.Y., Park H.S., Koo K.K. (2004). Behavior of Barium Acetate and Titanium Isopropoxide during the Formation of Crystalline Barium Titanate. Ind. Eng. Chem. Res..

[36] Lotnyk A., Senz S., Hesse D. (2006). Formation of BaTiO_3_ thin films from (110) TiO_2_ rutile single crystals and BaCO_3_ by solid state reactions. Solid State Ion..

[37] Yin H.M., Xu W.J., Zhou H.W., Zhao X.Y., Huang Y.N. (2019). Effects of phase composition and grain size on the piezoelectric properties of HfO_2_-doped barium titanate ceramics. J. Mater. Sci..

[38] Anwar S., Sagdeo P.R.R., Lalla N.P.P. (2006). Ferroelectric relaxor behavior in hafnium doped barium-titanate ceramic. Solid State Commun..

[39] Anwar S., Sagdeo P.R., Lalla N.P. (2006). Crossover from classical to relaxor ferroelectrics in BaTi_1−x_Hf_x_O_3_ ceramics. J. Phys. Condens. Matter.

[40] von Hippel A.R., Morgan S.O. (1955). Dielectrics and Waves. J. Electrochem. Soc..

[41] Kittel C., Masi J.F. (1954). Introduction to Solid State Physics. Phys. Today.

[42] Lethiecq M., Levassort F., Tran-Huu-Hue L.-P., Alguero M., Pardo L., Bove T., Ringgaard E., Wolny W. New low acoustic impedance piezoelectric material for broadband transducer applications. Proceedings of the IEEE Ultrasonics Symposium, 2004.

[43] CTS Ferroperm Piezoceramics. https://www.ferropermpiezoceramics.com/.

